# P-1492. Patient Characteristics Associated with Extended-Spectrum Beta-Lactamase-Producing Uropathogens: Pooled Results from Two Phase 3 Clinical Trials of Gepotidacin for the Treatment of Uncomplicated Urinary Tract Infection

**DOI:** 10.1093/ofid/ofae631.1662

**Published:** 2025-01-29

**Authors:** Jeremy Dennison, Amanda Sheets, Caroline R Perry, Florian Wagenlehner, Mark H Wilcox, Nicole E Scangarella-Oman, Deborah Butler, John Breton, Helen Millns, Aruni Mulgirigama, Matthew Helgeson, Salim Janmohamed

**Affiliations:** GSK, Brentford, UK, Brentford, England, United Kingdom; GSK, Collegeville, PA, USA, Collegeville, Pennsylvania; GSK, Collegeville, PA, USA, Collegeville, Pennsylvania; Justuf Liebeg University Diessen, Diessen, Hessen, Germany; LEEDS TEACHING HOSPITALS & UNIVERSITY OF LEEDS, LEEDS, England, United Kingdom; GlaxoSmithKline plc., Collegeville, Pennsylvania; GSK, London, England, United Kingdom; GSK, London, England, United Kingdom; GSK, Stevenage, UK, Stevenage, England, United Kingdom; GlaxoSmithKline plc., Collegeville, Pennsylvania; GSK, London, England, United Kingdom; GSK, Brentford, UK, Brentford, England, United Kingdom

## Abstract

**Background:**

Uncomplicated urinary tract infections (uUTIs), usually caused by *Escherichia coli* (*E. coli*), are common infections, affecting ∼50% of women globally in their lifetime. The presence of extended-spectrum beta-lactamase (ESBL)-producing Enterobacterales limits effective oral treatment options for uUTI. Using uropathogen susceptibility data from two randomized controlled trials (RCTs) in uUTI, we compared baseline characteristics of patients with ESBL-positive (ESBL+) versus non-ESBL uropathogens.

**Figure:**
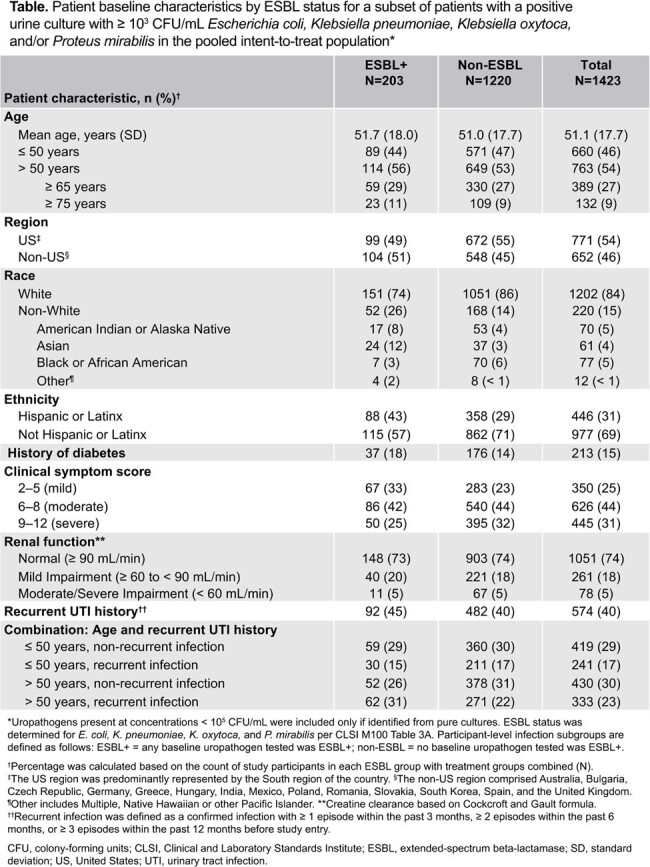

**Methods:**

EAGLE-2 (NCT04020341) and -3 (NCT04187144) were Phase 3, double-blind RCTs of oral gepotidacin versus nitrofurantoin for uUTI. Patients were female, aged ≥ 12 years, with ≥ 2 uUTI symptoms and urinary nitrite and/or pyuria. Pretreatment clean-catch midstream urine samples were collected for quantitative culture and susceptibility. In this post-hoc exploratory analysis, patients in the intent-to-treat population (all randomized patients), with ≥ 10^3^ colony-forming units/mL *E. coli*, *Klebsiella pneumoniae*, *Klebsiella oxytoca*, and/or *Proteus mirabilis*, were grouped by ESBL status of their uropathogen(s), per Clinical and Laboratory Standards Institute guidelines.

**Figure:**
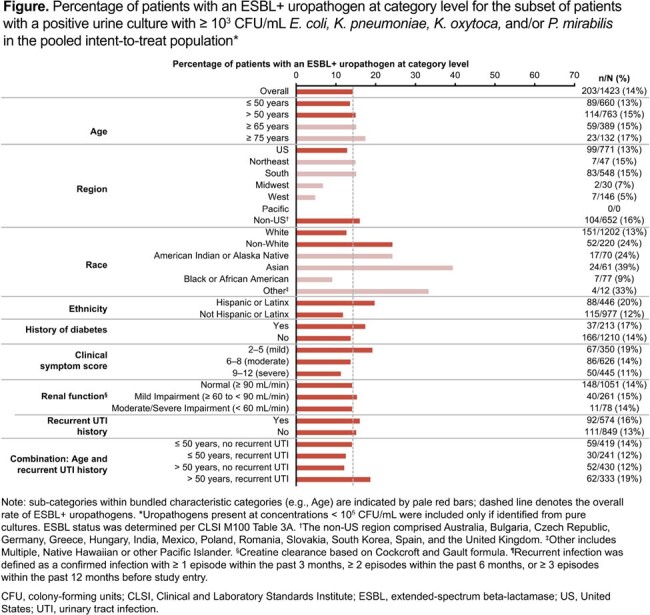

**Results:**

Overall, 1423 patients with culture-confirmed uUTI were included in the analysis; 203 (14%) had an ESBL+ uropathogen. Baseline characteristics by ESBL status are presented in the **Table**, and ESBL+ uropathogen prevalence by subgroup in the **Figure**. Generally, versus patients with non-ESBL uropathogens, more patients with ESBL+ uropathogens were: of Asian, American Indian or Alaska Native race (though sample sizes were small); of Hispanic/Latinx ethnicity; had milder symptoms; aged > 50 years with history of recurrent urinary tract infection.

**Conclusion:**

This post-hoc analysis of contemporary uUTI trial data describes baseline characteristics for patients with ESBL+ versus non-ESBL uropathogens. These data could inform future studies to identify clinical risk factors linked to ESBL+ uropathogens and improve patient outcomes.

**Funding:**

EAGLE-2 was funded in part by GSK and in part with Federal funds from the US Office of the Assistant Secretary for Preparedness and Response, Biomedical Advanced Research and Development Authority (HHSO100201300011C). EAGLE-3 was funded by GSK.

**Disclosures:**

**Jeremy Dennison, MD PhD**, GSK: Employee|GSK: Stocks/Bonds (Public Company) **Amanda Sheets, PhD**, GSK: Employee|GSK: Stocks/Bonds (Public Company) **Caroline R. Perry, PhD**, GSK: Employee|GSK: Stocks/Bonds (Public Company) **Florian Wagenlehner, MD**, Astellas: Advisor/Consultant|AstraZeneca: Advisor/Consultant|Bionorica: Advisor/Consultant|DFG (German Research Foundation) funded research group BARICADE (FOR5427/1-466687329): Speaker|GSK: Advisor/Consultant|GSK: Principal investigator in a GSK-sponsored study|Janssen: Advisor/Consultant|Klosterfrau: Advisor/Consultant|MIP Pharma: Advisor/Consultant|OM Pharma: Advisor/Consultant|Spero: Advisor/Consultant|VenatoRX: Advisor/Consultant **Mark H. Wilcox, MD**, Astra Zeneca: Advisor/Consultant|Debiopharm International S.A.: Advisor/Consultant|Debiopharm International S.A.: Grant/Research Support|Ferring: Advisor/Consultant|GSK: Advisor/Consultant|GSK: Honoraria|Nestle: Advisor/Consultant|Paion: Advisor/Consultant|Pfizer: Advisor/Consultant|Pfizer: Grant/Research Support|Pfizer: Honoraria|Phico therapeutics: Advisor/Consultant|QPex Biopharma: Advisor/Consultant|Seres: Advisor/Consultant|Seres: Grant/Research Support|Seres: Lecture Fees|Summit: Advisor/Consultant|Summit: Grant/Research Support|The European Tissue Symposium: Advisor/Consultant|The European Tissue Symposium: Grant/Research Support|Tillotts: Advisor/Consultant|Tillotts: Grant/Research Support|Tillotts: Lecture Fees|Vedanta: Advisor/Consultant **Nicole E. Scangarella-Oman, MS**, GSK: Employee|GSK: Stocks/Bonds (Public Company) **Deborah Butler, PharmD**, GSK: Employee|GSK: Stocks/Bonds (Public Company) **John Breton, MCM**, GSK: Employee|GSK: Stocks/Bonds (Public Company) **Helen Millns, PhD**, GSK: Employee|GSK: Stocks/Bonds (Public Company) **Aruni Mulgirigama, MBBS**, GSK: Employee|GSK: Stocks/Bonds (Public Company) **Matthew Helgeson, PharmD, MBA**, GSK: Employee|GSK: Stocks/Bonds (Public Company) **Salim Janmohamed, MD**, GSK: Employee|GSK: Stocks/Bonds (Public Company)

